# Fucoidan Is Not Completely Dependent on Degradation to Fucose to Relieve Ulcerative Colitis

**DOI:** 10.3390/ph15040430

**Published:** 2022-03-31

**Authors:** Qiang Wei, Maochen Xing, Ke Wang, Qiong Yang, Jiarui Zhao, Yuan Wang, Xia Li, Kai Ji, Shuliang Song

**Affiliations:** 1 Marine College, Shandong University, Weihai 264209, China; wq18086420598@163.com (Q.W.); sddxxmc@163.com (M.X.); wk1308866506@163.com (K.W.); yangqiong1237@163.com (Q.Y.); 201936684@mail.sdu.edu.cn (J.Z.); wangyuan1126@mail.sdu.edu.cn (Y.W.); xiali@sdu.edu.cn (X.L.); 2 Department of Plastic Surgery, China–Japan Friendship Hospital, Beijing 100029, China; 3 Weihai Research Institute of Industrial Technology, Shandong University, Weihai 264209, China

**Keywords:** fucoidan, fucose, ulcerative colitis, gut microbiota, dextran sulfate sodium

## Abstract

Recently, fucoidan has been proposed for use as a potential anti-inflammatory drug. The purpose of this study was to investigate the mechanism of fucoidan in the treatment of ulcerative colitis. We compared the anti-inflammatory effects of fucoidan and fucose induced by dextran sulfate sodium, and the effects of fucoidan and fucose on the gut microbiota of mice. Our results showed that low-dose fucoidan significantly improved weight loss, disease activity index scores, colonic shortening, colonic histopathological damage, intestinal fatty acid binding protein 2 levels, and the expression of Occludin, Claudin-4, and Claudin-1. However, both high-dose fucoidan and fucose did not perform as well as low-dose fucoidan as described above. In addition, 16S rDNA high-throughput sequencing showed that low-dose fucoidan significantly increased the abundance of *Alloprevotella*, and fucose significantly increased *Ruminococcaceae*, but neither significantly reversed the imbalance in the gut microbiota. Therefore, we inferred that the regulation of fucoidan on colitis has a unique and complex mechanism, and it is not completely dependent on degradation to fucose to relieve ulcerative colitis, nor is it achieved only by regulating the gut microbiota. The mechanism by which fucoidan treats colitis may also include reducing inflammatory cell infiltration and increasing intestinal barrier function.

## 1. Introduction

Ulcerative colitis (UC) is a chronic inflammation of the colon with a complexity of causes, and it can have a devastating effect on colon health [1]. In recent years, the number of UC cases among humans has been increasing.

Fucoidan is usually derived from the cell walls of brown algae and some marine invertebrate tissues and exhibits many biological activities suitable for therapeutic applications [2,3], including anti-inflammatory [4], anti-oxidation [5], immune regulation [6], and anti-tumor [7] effects. Studies have found that fucoidan can improve dextran sulfate sodium (DSS)-induced weight loss in pigs, as well as diarrhea scores and changes in colitis-related clinical indicators of patients under the influence of DSS [8]. Therefore, the anti-inflammatory effects of fucoidan in the treatment of UC are worthy of attention.

Although the exact pathological mechanism of UC is still unclear, it is generally believed that destruction of the epithelial barrier leads to invasion of the mucosal layer by bacterial antigens, which activate the pathogenic mucosal immune system, confuse the immune response, and eventually attack the colon [9]. A study found that fucoidan can attenuate DSS-induced colonic mucosal damage and crypt destruction in mice [10]. In another study, fucoidan improved intestinal mucosal barrier function in nonobese diabetic mice by increasing the expression of tight-junction-related proteins in the gut [11]. In addition, fucose reduces colitis severity by inhibiting macrophage M1 polarization, inhibiting the NOD-like receptor family pyrin domain containing 3 (NLRP3) inflammasome and Nuclear factor-kappa B (NF-KB) activation, and down-regulation of pro-inflammatory cytokines [12]. Therefore, it is necessary to further study whether fucoidan plays a role in the treatment of UC mice by degradation to fucose.

The gut microbiota encompasses a large number of microorganisms that settle on the surface of the intestinal mucosa and in the intestinal cavity [13]. Many genetic studies have emphasized the role of host-microbial interactions in UC pathogenesis [14,15]. It should be noted that fucoidan, as a highly sulfated macromolecule, is rarely absorbed by the intestinal mucosa [16]. Whether fucoidan can be metabolized by gut microbiota remains controversial [17]. Therefore, the effects of fucoidan and fucose on gut microbiota need to be further investigated.

DSS-induced colitis in mice is a common UC model, which is associated with severe intestinal epithelial injury and strong inflammatory response in the colon, and its key clinical and pathological features are similar to humans [18]. Rakoff et al. demonstrated that the interaction between host innate immunity and gut microbes is the key to the mechanism of DSS-induced intestinal pathogenesis [19]. In this study, DSS was used to establish the UC model to study the anti-inflammatory effects of fucoidan and fucose. The effect of fucoidan and fucose on the colon microbiota of UC mice was analyzed using 16S rDNA high-throughput sequencing.

## 2. Results

### 2.1. Fucoidan Alleviated Weight Loss and Decreased the DAI Score Better Than Fucose

**Figure 1:**
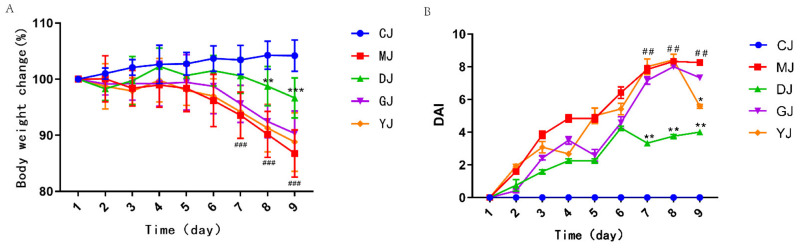
(**A**) The effect of fucoidan and fucose on the weight change rate of UC mice. (**B**) The effect of fucoidan and fucose on the DAI score of UC mice. ## *p* < 0.01, ### *p* < 0.001 vs. the CJ group; * *p* < 0.05, ** *p* < 0.01, *** *p* < 0.001 vs. the MJ group. (CJ: control group, MJ: model group, DJ: low-dose fucoidan group, GJ: high-dose fucoidan group, YJ: fucose group).

As shown in Figure 1A, the body weight of the CJ group gradually increased with food intake, and the body weight of the other groups decreased with the intervention of DSS from days 5 to 9. In addition, the body weight change of the DJ group was significantly higher than those of the MJ group (*p* < 0.01), the GJ group, and the YJ group on the 7th day, the 8th day, and the 9th day, respectively. Therefore, the DJ group could significantly alleviate the weight loss in mice with colitis, and the effect was better than those of the GJ group and the YJ group.

As the modeling time progressed, the DAI score of the model group increased (*p* < 0.05) (Figure 1B). On the 6th day, blood was observed in the feces, and symptoms such as watery stool and mental atrophy appeared. During the first six days, it was clear that the DAI score increased progressively in all groups except the control group because of the intervention of DSS. From days 7 to 9, DAI line plots in the low-dose fucoidan group were significantly flatter and lower than those in the other groups. However, compared with the model group, the high-dose fucoidan group and the fucose group did not improve the DAI score well, which may be related to the dose. Therefore, low-dose fucoidan can effectively reduce the DAI score in mice with colitis, the dose of fucoidan is not as high as possible, and there may be a suitable drug dose range.

### 2.2. Fucoidan Alleviated Colon Shortening Better Than Fucose

**Figure 2:**
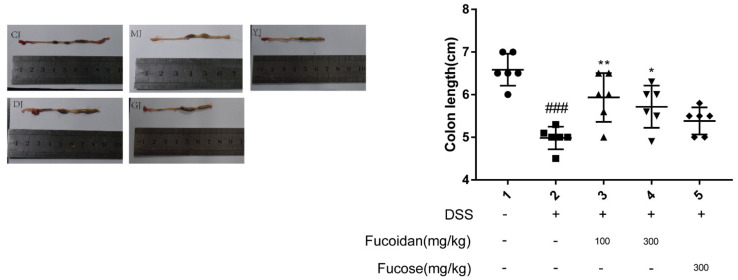
The effect of fucoidan and fucose on colon length in mice. ### *p* < 0.001 vs. the CJ group; * *p* < 0.05, ** *p* < 0.01, vs. the MJ group. (CJ: control group, MJ: model group, DJ: low-dose fucoidan group, GJ: high-dose fucoidan group, YJ: fucose group).

We further evaluated the effect of fucoidan and fucose on colon length in mice. As shown in Figure 2, the colon length in the model group was significantly shorter than that in the CJ group (*p* < 0.01). Colons in the DJ group and the GJ group were significantly longer than those in the MJ group after fucoidan treatment. In addition, both low and high doses were more efficacious than fucose. Therefore, both high and low doses of fucoidan can effectively alleviate colon shortening in mice with colitis, and the effect is better than that of fucose.

### 2.3. Fucoidan Reduced IFABP2 in Serum Better Than Fucose

**Figure 3:**
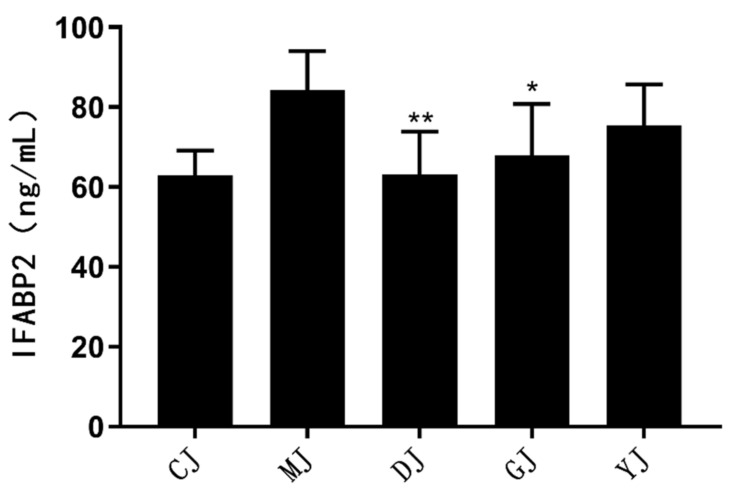
The effects of fucoidan and fucose on the concentration of IFABP2 in serum of mice. * *p* < 0.05, ** *p* < 0.01, vs. the MJ group. (CJ: control group, MJ: model group, DJ: low-dose fucoidan group, GJ: high-dose fucoidan group, YJ: fucose group).

To investigate the effect of fucoidan on intestinal permeability of mice, the concentration of intestinal fatty acid binding protein 2 (IFABP2) in the blood of the mice was detected using an ELISA kit. As shown in Figure 3, IFABP2 levels were higher in the MJ group than in the CJ group. The IFABP2 concentration in the blood of mice in the DJ group was significantly lower than that in the MJ group (*p* < 0.01), and IFABP2 concentrations in the DJ group were lower than those in the GJ and the YJ group. This indicated that the mechanical barrier in the model mice colons might have been damaged, and low-dose fucoidan may help maintain the normal permeability of the colon.

### 2.4. Fucoidan Alleviated Colon Histopathology Injury Better Than Fucose

**Figure 4:**
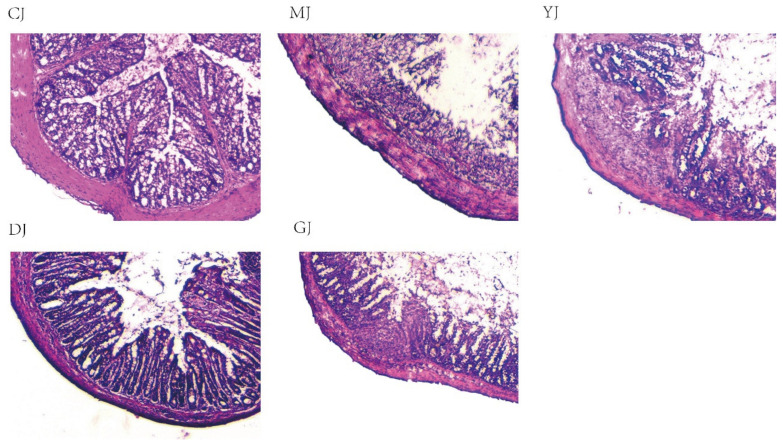
Hematoxylin and eosin (H&E) staining of colon tissue section in each group of mice (200×). (CJ: control group, MJ: model group, DJ: low-dose fucoidan group, GJ: high-dose fucoidan group, YJ: fucose group).

The colon of the control group was found to be of normal shape, with complete intestinal epithelium and orderly crypt arrangement, and contained a large number of goblet cells (Figure 4). Almost no inflammatory cell infiltration was found in the control group. After DSS intervention, a large number of mucosal epithelial cells were denatured and necrotic, inflammatory cells had infiltrated the mucosa and submucosa, goblet cell numbers were reduced, and the intestinal crypt was damaged. After low-dose fucoidan intervention, colonic mucosa ulcers and erosion were obviously reduced or disappeared, inflammatory cell infiltration was reduced, goblet cell and gland arrangement were restored, and pathological injury to the colonic mucosa was significantly improved. In addition, the GJ and the YJ group showed improvements to the colonic mucosa injury to some extent, but some epithelial and crypt structures were damaged, and inflammatory infiltration could be seen. These are consistent with the effects of the DJ group and the GJ group on the DAI score in UC mice. Overall, the protective effect of low-dose fucoidan on the colonic mucosa of UC mice was superior to the other treatments.

### 2.5. Fucoidan Increased the Expression of Tight-Junction-Related mRNA and Proteins Better Than Fucose

**Figure 5:**
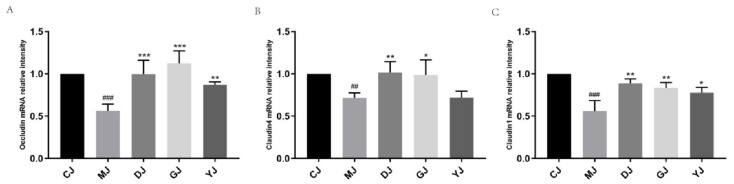
Effect of fucoidan and fucose on the expression of tight-junction-related mRNA in mice with colitis. (**A**–**C**) The relative gene expression of Occludin, Claudin-4, and Claudin-1 was detected by qPCR, and the data were generated from colon tissues., ## *p* < 0.01, ### *p* < 0.001 vs. the CJ group; * *p* < 0.05, ** *p* < 0.01, *** *p* < 0.001 vs. the MJ group. (CJ: control group, MJ: model group, DJ: low-dose fucoidan group, GJ: high-dose fucoidan group, YJ: fucose group).

Due to the increased intestinal permeability and colon histopathology injury in UC mice, we further evaluated the expression of tight-junction-related mRNA in the colon (Figure 5). Compared with the CJ group, the expression levels of Occludin, Claudin-4, and Claudin-1 in the MJ group were significantly lower than those in the control group. The results showed that the intestinal barrier of mice was damaged to some extent after DSS intervention. After treatment with fucoidan, the mRNA expression of Occludin, Claudin-4, and Claudin-1 increased significantly, and the effects seen in the DJ and GJ groups were better than those in the YJ group, in which only Claudin-1 and Occludin were increased.

**Figure 6:**
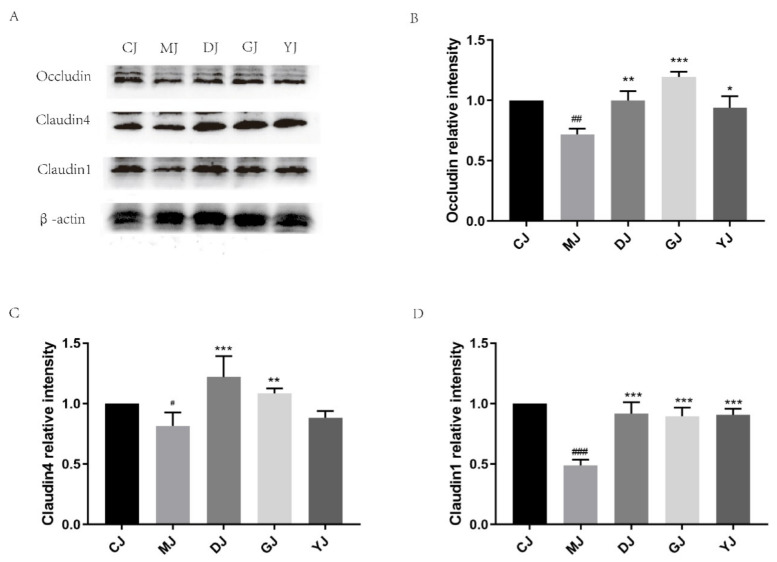
Effects of fucoidan and fucose on the expression of tight-junction-related proteins. (**A**) Representative immunoblot bands for the Occludin, Claudin-4, and Claudin-1 proteins. β-actin was used as loading control. (**B**–**D**) Histogram of relative expression of Occludin, Claudin-4, and Claudin-1. # *p* < 0.05, ## *p* < 0.01, ### *p* < 0.001 vs. the CJ group; * *p* < 0.05, ** *p* < 0.01, *** *p* < 0.001 vs. the MJ group. (CJ: control group, MJ: model group, DJ: low-dose fucoidan group, GJ: high-dose fucoidan group, YJ: fucose group).

The Western blot showed the expression of tight-junction-related proteins in the colon (Figure 6). Compared with the control group, the expression of Occludin, Claudin-4, and Claudin-1 decreased significantly in the model group, which is consistent with the corresponding mRNA expression levels. After treatment with fucoidan, the expression of Occludin, Claudin-4, and Claudin-1 increased significantly. However, fucose only increased the expression of Claudin-1 and Occludin. Therefore, fucoidan was superior to fucose in enhancing the expression of tight-junction-related proteins in UC mice.

### 2.6. The Effect of Fucoidan and Fucose on Gut Microbiota

#### 2.6.1. Fucoidan and Fucose Modulated the Structure and Diversity of the Gut Microbiota

**Figure 7:**
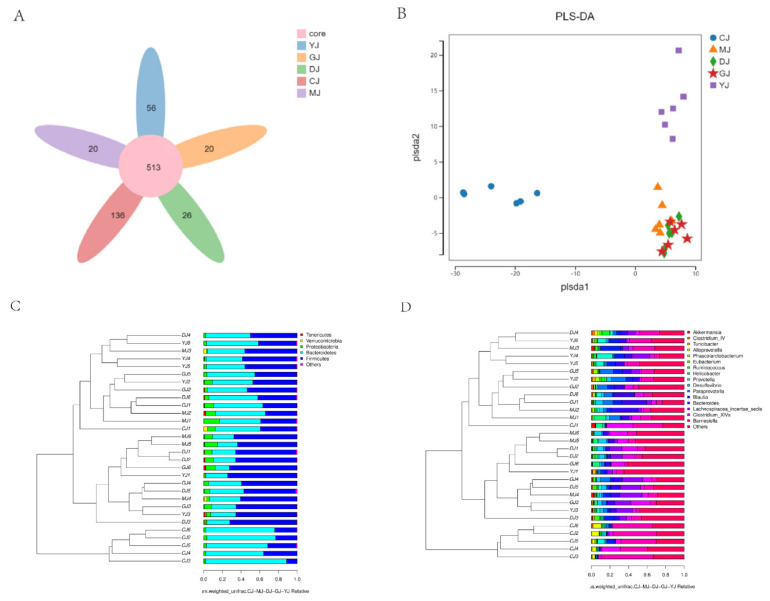
Fucoidan and fucose modulated the structure and diversity of the gut microbiota. (**A**) OTU Core-Pan diagram; (**B**) PLS-DA-reduced dimension diagram; (**C**) UPGMA cluster diagram at the phylum level; (**D**) UPGMA cluster diagram at the genus level. (CJ: control group, MJ: model group, DJ: low-dose fucoidan group, GJ: high-dose fucoidan group, YJ: fucose group).

The OTU Core-Pan diagram showed that there were 771 OTUs in the colons of the five groups of mice, 513 of which were in all groups (Figure 7A). There were 649 OTUs in the CJ group, 569 OTUs in the YJ group, 539 OTUs in the DJ group, and 533 OTUs in the MJ group and the GJ group. The results showed that the number of OTUs in the Model group decreased significantly after DSS administration. Both low-dose fucoidan and fucose alleviated the decrease in OTUs in UC mice, and fucose was the more effective of the two.

PLS-DA analysis showed that the structure of the gut microbiota along PLS-DA1 was significantly changed after DSS intervention (Figure 7B). After treatment with fucoidan and fucose, the structure of PLS-DA1 remained unchanged, which may be because fucoidan and fucose did not affect the structure of the PLS-DA1. In addition, fucose induced a change in the gut microbiota along PLS-DA2 in the Model group. Therefore, fucoidan and fucose may have a therapeutic impact by affecting the structure of the gut microbiota.

UPGMA cluster analysis based on the weighted unifrac distance matrix (Figure 7C,D), whether at the gate or genus level, showed two main clusters: the control group (except CJ1) and a cluster containing the other groups. The results showed that the structure of the gut microbiota changed significantly after DSS intervention.

#### 2.6.2. Effect of Fucoidan and Fucose on Species Composition of Gut Microbiota

##### Relative Abundance of the Dominant Bacteria in Different Groups at Phylum and Genus Levels

**Figure 8:**
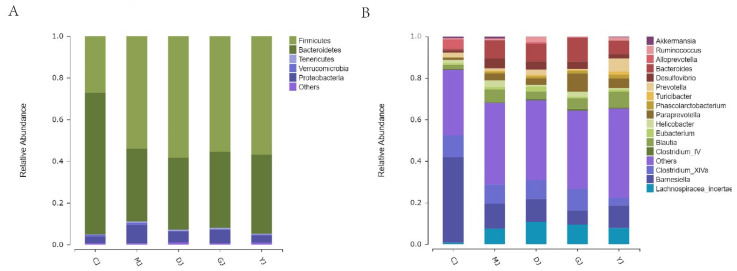
Relative abundance of the dominant bacteria in different groups at phylum and genus levels. (**A**) Relative abundance of coliforms at the phylum level; (**B**) relative abundance of coliforms at the genus level. (CJ: control group, MJ: model group, DJ: low-dose fucoidan group, GJ: high-dose fucoidan group, YJ: fucose group).

At the phylum level (Figure 8A), the structure of microbiota in each group was similar, but the proportion of microbiota differed. The results of Zhang et al. [20] are consistent with those for the dominant microbiota, which included Firmicutes, Bacteroidetes, and Proteobacteria.

At the genus level (Figure 8B), the differences between each group were large, indicating that DSS, fucoidan, and fucose all had a large influence on the intestinal microbiota of the UC mice.

##### LEfSe Analysis Identified the Most Differentially Abundant Taxons between the Two Groups

**Figure 9:**
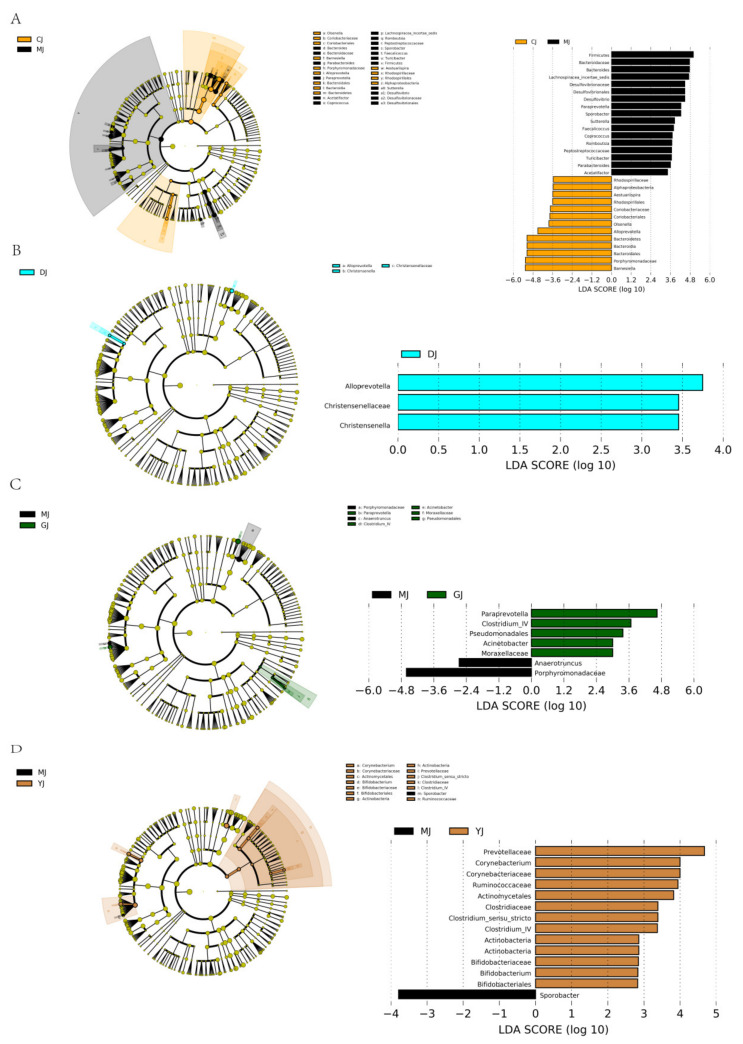
LEfSe analysis identified the most differentially abundant taxons between the two groups. (**A**) LEfSe analysis of gut microbiota between CJ group and MJ group; (**B**) LEfSe analysis of gut microbiota between MJ group and DJ group; (**C**) LEfSe analysis of gut microbiota between MJ group and GJ group; (**D**) LEfSe analysis of gut microbiota between MJ group and YJ group. (All *p* < 0.05, shown only when the absolute value is greater than 2). (CJ: control group, MJ: model group, DJ: low-dose fucoidan group, GJ: high-dose fucoidan group, YJ: fucose group).

At the phylum level (Figure 9A–D), after DSS intervention, the abundance of Bacteroidetes decreased significantly and that of Firmicutes increased significantly in the model group. A decrease or increase in the abundance ratio of Bacteroidetes/Firmicutes (B/F) was found to be an indicator of microbial imbalance. The B/F of the model group was significantly decreased, which indicated that the colitis induced by DSS resulted in a severe imbalance of gut microbiota in the mice. Unfortunately, neither fucoidan nor fucose reversed the changes to the population abundances of Firmicutes and Bacteroidetes.

The columnar abundance map of the gut microbiota can be used to explore the content and distribution of intestinal microbiota among groups, but it does not reflect differences in the gut microbiota among groups; thus, in this study, we used LEFSE LDA analysis to find statistically different biomarkers among the groups.

At the genus level (Figure 9A–D), after DSS intervention, the abundance of *Desulfovibrio*, *Paraprevotella*, *Parabacteroides*, *Romboutsia,* and *Turicicter* all increased significantly, while markedly decreasing those of *Alloprevotella* and *Barnesiella*. The abundance of *Alloprevotella* increased significantly in the low-dose fucoidan group, and those of *Paraprevotella* and *Clostridium-IV* increased significantly in the high-dose fucoidan group. In addition, the abundance of *Ruminococcaceae* and *Clostridium-IV* increased significantly in the fucose group.

## 3. Discussion

Both fucoidan and fucose have been shown to have anti-inflammatory effects, but the mechanisms behind these effects are still unclear. Therefore, we studied the therapeutic effects of fucoidan and fucose on UC mice and analyzed their effects on the colon microbiota of UC mice via 16S rDNA high-throughput sequencing.

Weight loss, DAI scores, and shortened colons are considered to be the important indicators of clinical activity in colitis [21]. After intervention with fucoidan and fucose, weight loss, DAI score, and colon shortening were significantly improved in UC mice. In addition, using H&E staining, we observed reduced inflammatory cell infiltration and lower numbers of goblet cells and glands in UC mice after fucoidan intervention, significantly improving the pathological injury to the colonic mucosa, and the therapeutic effect was better than that of fucose. These results suggest that fucoidan has a better therapeutic effect than fucose in alleviating DSS-induced colitis. It was concluded that fucoidan does not seem to be completely degraded to fucose in the treatment of UC.

IFABP2 is a kind of intestinal fatty acid binding protein. When the intestinal mucosa is damaged, IFABP2 is released into the blood; therefore, the concentration of IFABP2 in serum can be used as an index to evaluate the severity of colonic inflammation [22]. The results showed that the concentration of IFABP2 in serum decreased significantly after treatment with fucoidan, but there was no significant difference in serum IFABP2 after fucose was administered. Therefore, fucoidan may reduce the release of IFABP2 into the blood by repairing intestinal mucosal damage.

As inflammation is closely related to the formation of reactive intermediates (including reactive oxygen species and nitrogen compounds), oxidative stress is recognized as a fundamental mechanism of the pathophysiology of UC [23] and may lead to the destruction of the epithelial barrier, impairing tight-junction complexity, with a particularly potent impact on the Claudins [24,25,26]. Occludin also plays a complex role in the regulation of epithelial cells and immune homeostasis and is usually down-regulated in UC patients and DSS-induced colitis mouse models [24]. The effects of fucoidan and fucose on tight-junction-related genes and proteins in the mouse colon were studied by qPCR and Western blotting. The results showed that the expression levels of Occludin, Claudin-4, and Claudin-1 decreased significantly under the influence of DSS, while fucoidan significantly increased the expression levels of Occludin, Claudin-4, and Claudin-1, with a better effect than fucose, which is consistent with previous studies’ findings [11]. Moreover, the protective effect of fucoidan on the intestine of UC mice does not depend entirely on the activity of fucose.

In vitro, fucoidan promoted the growth of three lactic acid bacteria in the sugar-free medium [27]. The results indicated that fucoidan could be used as a carbon source by some lactic acid bacteria of the gut microbiota. Therefore, we further investigated the effects of fucoidan and fucose on the colon microbiota of UC mice. The results of the OTU statistical analysis, PLS-DA dimensionality reduction analysis, and UPGMA cluster analysis showed that DSS modeling significantly affected OTU abundance and the microbial community structure in each group, and fucoidan and fucose can affect the structure of the gut microbiota to a certain extent.

A decrease or increase in the abundance ratio of Bacteroidetes/Firmicutes (B/F) was found to be an indicator of microbial imbalance [28]. After DSS intervention, the abundance of Bacteroidetes decreased significantly, and the abundance of Firmicutes increased significantly in the model group; thus, B/F decreased significantly. The results showed that DSS-induced colitis resulted in a severe imbalance of gut microbiota in mice.

Short-chain fatty acids (SCFAs), one of the most abundant metabolites of gut microbiota in the colon, are mainly composed of acetic acid, propionic acid, and butyric acid [29]. They are produced by the fermentation of undigested food by microorganisms and are considered beneficial to human intestinal health [30]. Studies have shown that SCFAs have anti-inflammatory and anti-cancer effects and play important roles in maintaining the metabolic dynamic equilibrium of colon cells and protecting colon cells from external injury [31]. SCFAs can also enhance intestinal barrier function by promoting mucus production and increasing tight-junction expression [32,33].

*Alloprevotella* is an SCFA-producing genus whose abundance is negatively associated with obesity, diabetes, and metabolic syndrome, and these bacteria can exert anti-inflammatory effects [34,35,36]. Compared with the model group, the low-dose fucoidan group had significantly increased numbers of *Alloprevotella*. Therefore, low-dose fucoidan may be used to treat colitis inflammation by increasing the abundance of *Alloprevotella* species in the colon.

*Ruminococcaceae*, a family of autochthonous and benign species, found mainly in the cecum and colon, increase the production of SCFAs and play an important role in the degradation of various polysaccharides and fibers [37], including starch, cellulose, and xylan [38,39]. *Ruminococcaceae* are negatively correlated with an increase in intestinal permeability. After intervention with fucose, the abundance of *Ruminococcaceae* was significantly increased. Therefore, fucose may increase the abundance of *Ruminococcaceae* in the colon to treat colitis.

Although no specific pathogens of UC have been found, dysbacteriosis of the gut microbiota may induce or aggravate the development of inflammatory bowel disease [40]. The results showed that fucoidan and fucose did not significantly modulate the dysbacteriosis of *Firmicutes*, *Bacteroides*, *Desulfovibrio*, *Paraprevotella*, *Parabacteroides*, *Romboutsia*, *Turibacter,* and *Barnesiella* induced by DSS. The high-dose fucoidan group and fucose group were also found to cause a significant increase in *Clostridium-IV*, which belongs to the genus *Clostridium* and is often thought to increase inflammation and carcinogenicity in the gut [41]. Therefore, the dose of fucoidan is not as high as possible, and there may be a suitable drug dose range.

## 4. Materials and Methods

### 4.1. Animals and Treatment

C57BL/6 male mice (Daren Fortune Animal Technology, Qingdao, China) at 4 weeks old were bred at a constant temperature (24 ± 2 °C) and constant humidity (40–60%). All mice had free access to autoclaved water and the same batch of standard laboratory diet to minimize the variation in environmental factors. The study was conducted according to the guidelines of the Declaration of Helsinki and approved by the Ethics Committee of Shandong University Weihai Municipal Hospital (Permission ID: 2021084). The fucoidan extracted from the brown alga Fucus vesiculosus was purchased from Rizhao Jiejing Marine Biotechnology Development Co., Ltd., Rizhao, China, containing fucose (38%), sulfate radical (20%), uronic acid (9%), and minor amounts of amino sugar and protein, as well as a peak molecular weight of 170KDa, as assessed using multi-angle laser light scattering. Fucose was purchased from Aladdin, China. DSS (MW 36 KDa–50 KDa; MP Biomedicals, Irvine, CA, USA) was dissolved in distilled water to prepare a 2.5% (*w*/*w*) solution. All mice had free access to drinking water and the same batch of standard laboratory diet to minimize the variation in environmental factors.

After a one-week adaptation, mice were randomly divided into five groups (*n* = 6 per group): (1) control group (CJ), (2) model group (MJ), (3) low-dose fucoidan group (DJ), (4) high-dose fucoidan group (GJ), (5) fucose group (YJ). Acute colitis was induced by administration of 2.5% (*w*/*w*) DSS in drinking water for one DSS cycle (7 consecutive days) and then followed by 3 days with normal drinking water. Low-dose fucoidan (100 mg/kg), high-dose fucoidan (300 mg/kg), and fucose (300 mg/kg), dissolved in drinking water, were given to mice by gavage once a day for 9 days. In the control and model group, the same volume of drinking water was added by gavage as a vehicle control. All mice were sacrificed on day 10. The establishment of the acute ulcerative colitis model and intervention therapy with drugs is shown in Figure S1 (Supplementary Materials).

During the experiment, we observed and recorded the body weight, stool consistency, and stool bleeding (Fecal Occult Blood Testing; ELISA Kit, Zhuhai Baso Biotechnology Co., Ltd., Zhuhai, China) of the mice every day. On the 10th day, after enucleation, serum was collected, centrifuged at 3000 r/min for 10 min (5810R, Eppendorf, Hamburg, Germany), and cryopreserved. The mice were anesthetized with diethyl ether and then sacrificed, and the colon tissue samples and their contents were collected and stored separately. All of the above sampling procedures were carried out on a sterile operating table, and all samples were stored in a freezer at −80 °C.

### 4.2. DAI Scores

After collecting data from the samples, the disease activity index (DAI) was calculated based on the average weight loss, stool consistency, and stool bleeding [42]. The DAI scores are shown in Table S1 (Supplementary Materials). DAI scores = weight loss + stool consistency + stool bleeding.

### 4.3. IFABP2 Measurements

The concentration of intestinal fatty acid binding protein 2 (IFABP2) in the serum of mice was measured by an ELISA kit (Baolaibo, Beijing, China) according to the manufacturer’s manuals. The absorbance was obtained at a relative nanometer wavelength using a microplate reader (Biotek Instruments, Inc., Winooski, VT, USA).

### 4.4. Histological Analysis

The colon tissues of each group were placed on the loading table of a Microtome Cryostat (Leica CM1860UV, Weztlar, Germany), and a cryosectioning embedding agent (Biosharp, Hefei, China) was injected around the samples to completely immerse them. After about 20 min, the embedding agent had fixed the sample. The sections were cut to 5 μm thickness and fixed on slides in a fixative solution (Beyotime, Shanghai, China) for more than 10 min, and washed with distilled water for 2 min. The hematoxylin and eosin (H&E) staining kit (Beyotime, Shanghai, China) was used to stain the tissue sections. Finally, an inverted light microscope (OLYMPUS CKX53, Tokyo, Japan) was used to observe the sections and take pictures.

### 4.5. RNA Extraction and Quantitative Real-Time PCR (qRT-PCR)

Total RNA was extracted with Trizol reagent (Sangon Biotech, Shanghai, China) and reverse-transcribed in the presence of oligomeric primers (0.2 μL each). Messenger RNA (mRNA) was quantified by SYBR Green Kit (Spark Jade, Jinan, China) and amplified by a PCR instrument (Bio-Rad, Power PacBasic, California, CA, USA) according to the manufacturer’s protocol. The expression level of the relative genes was normalized relative to levels of the housekeeping gene β-actin and calculated by using the comparative cycle threshold (2 − ΔΔCt) method. The primer sequences are shown in Table S2 (Supplementary Materials).

### 4.6. Western Blot Analysis

Proteins were harvested from colon tissues with RIPA Lysis Buffer (Beyotime, Shanghai, China) supplemented with phenylmethylsulfonyl fluoride (PMSF, Thermo Scientific, Massachusetts, MA, USA) protease inhibitor. Total protein content in the supernatant was determined by the BCA protein assay kit (Beyotime, Shanghai, China); denatured protein samples of an appropriate quality of proteins were subjected to sodium dodecyl sulfate-polyacrylamide gel electrophoresis (SDS-PAGE) and then electrotransferred to a PVDF membrane (Sigma, Missouri, MO, USA); and proteins on the membrane were immunodetected with specific antibodies for rabbit anti-Occludin (Wanleibio, Shenyang, China), rabbit anti-Claudin-4 (Abbkine, San Diego, CA, USA), rabbit anti-Claudin-1 (Wanleibio, Shenyang, China), and β-actin (ZSGB-BIO, Beijing, China) overnight at 4 °C. Protein bands were visualized by the enhanced chemiluminescence (ECL) kit (Beyotime, Shanghai, China).

### 4.7. 16S rDNA Gene High-Throughput Sequencing

In each group, 30 ng of colon contents were mixed with the corresponding primers to prepare the PCR reaction system. The PCR products were purified with Agencourt AMPure XP magnetic beads, dissolved in the kit elution buffer, and the library was constructed. The Agilent 2100 Bioanalyzer was used to detect the fragment sizes and concentration of the library. The HiSeq platform was selected to sequence the qualified libraries according to the sizes of the insert fragments.

### 4.8. Statistical Analysis

Statistical analysis was performed using the SPSS, Graph-pad PRISM, R (v3.1.1), R (v3.2.1), phytools, R (v3.5.1), R (v3.4.1), and LEfSe software. Data are presented as mean ± SEM, and multiple group comparisons were performed using one-way analysis of variance (ANOVA) and then tested with Tukey’s multiple comparisons test. *p* < 0.05 was considered statistically significant.

## 5. Conclusions

The present study showed that low-dose fucoidan improved weight loss, DAI score, colon shortening, IFBP2 concentration, and histopathological damage, and increased tight-junction-related proteins and mRNA in the colon. However, both high-dose fucoidan and fucose did not perform as well as low-dose fucoidan as described above. Therefore, fucoidan was not completely degraded to fucose to treat ulcerative colitis. In addition, the results of the gut microbiota showed that the regulation effect of fucoidan on most gut microbiota was not very significant, especially high-dose fucoidan, and it could not improve most of the gut microbiota imbalance, even leading to the increase in some harmful bacteria. This shows that when fucoidan is used in the treatment of colitis, a higher dose is not better, and the optimal dose needs to be further explored. In conclusion, the treatment of fucoidan on colitis has a unique and complex mechanism, and it is not completely degraded to fucose to relieve ulcerative colitis, nor is it achieved only by regulating the gut microbiota. The mechanism by which fucoidan treats colitis may also include reducing inflammatory cell infiltration and increasing intestinal barrier function.

## Data Availability

Data is contained within the article and Supplementary Materials.

## Supplementary Materials

The following supporting information can be downloaded at: https://www.mdpi.com/article/10.3390/ph15040430/s1, Figure S1: The establishment of acute ulcerative colitis model and intervention therapy with drugs; Table S1: Disease Activity Index of mice (DAI rating scale); Table S2: PCR primers.

## Abbreviations

UC: ulcerative colitis; DSS: dextran sulfate sodium; IFABP2: intestinal fatty acid binding protein 2; NLRP3: NOD-like receptor family pyrin domain containing 3; NF-κ B: Nuclear factor-kappa B; H&E: hematoxylin and eosin
