# Supplementary material for: Fucoidan Is Not Completely Dependent on Degradation to Fucose to Relieve Ulcerative Colitis
Source: Pharmaceuticals (Basel). 2022 Mar 31;15(4):430. doi: 10.3390/ph15040430 (PMC9030999; doi:10.3390/ph15040430)
Supplement: Supplementary file 1 [file pharmaceuticals-15-00430-s001.zip › pharmaceuticals-1618892-supplementary.pdf]

Supplementary Material

Figure S1 Establishment of acute ulcerative colitis model and intervention therapy with drugs

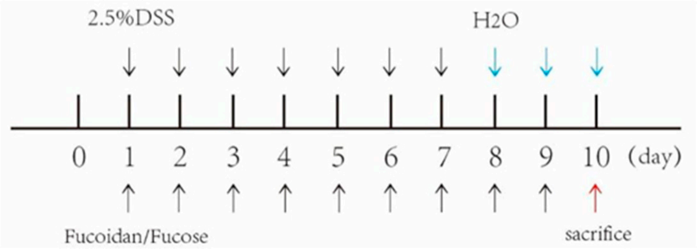

Table S1 Disease Activity Index of mice (DAI rating scale)

**Score**	**Weight loss**	**stool consistency**	**stool bleeding**
0	＜1%	normal	normal
1	1%-5%	fluffy but tangible	weakly positive
2	6%-10%	loose stool	positive
3	11%-18%	very loose and moist	Visible blood
4	＞18%	diarrhea	gross blood

Table S2 PCR primers

**Genes**	**Sequence 5'-3'**	**Tm （℃）**
Claudin4	Forward: TTATGGTCATCAGCATCATCGT	53
	Reverse:TGATCATGATCTTGGCCTTGAC	54
Occludin	Forward:TGCTTCATCGCTTCCTTAGTAA	53
	Reverse:GGGTTCACTCCCATTATGTACA	53
Claudin1	Reverse: AGATACAGTGCAAAGTCTTCGA	53
	Forward: CAGGATGCCAATTACCATCAAG	53
β -actin	Forward: CTACCTCATGAAGATCCTGACC	54
	Reverse: CACAGCTTCTCTTTGATGTCAC	53
